# Association of jasmonic acid priming with multiple defense mechanisms in wheat plants under high salt stress

**DOI:** 10.3389/fpls.2022.886862

**Published:** 2022-08-16

**Authors:** Mohamed S. Sheteiwy, Zaid Ulhassan, Weicong Qi, Haiying Lu, Hamada AbdElgawad, Tatiana Minkina, Svetlana Sushkova, Vishnu D. Rajput, Ali El-Keblawy, Izabela Jośko, Saad Sulieman, Mohamed A. El-Esawi, Khaled A. El-Tarabily, Synan F. AbuQamar, Haishui Yang, Mona Dawood

**Affiliations:** ^1^College of Biology and the Environment, Nanjing Forestry University, Nanjing, China; ^2^Department of Agronomy, Faculty of Agriculture, Mansoura University, Mansoura, Egypt; ^3^Southern Federal University, Academy of Biology and Biotechnology, Rostov-on-Don, Russia; ^4^Institute of Crop Science and Zhejiang Key Laboratory of Crop Germplasm, Zhejiang University, Hangzhou, China; ^5^Institute of Agriculture Resources and Environment, Jiangsu Academy of Agricultural Sciences (JAAS), Nanjing, China; ^6^Co-innovation Center for the Sustainable Forestry in Southern China, Nanjing Forestry University, Nanjing, China; ^7^Department of Botany, Faculty of Science, University of Beni-Suef, Beni-Suef, Egypt; ^8^Department of Applied Biology, Faculty of Science, University of Sharjah, Sharjah, United Arab Emirates; ^9^Faculty of Agrobioengineering, Institute of Plant Genetics, Breeding and Biotechnology, University of Life Sciences, Lublin, Poland; ^10^Department of Agronomy, Faculty of Agriculture, University of Khartoum, Khartoum North, Sudan; ^11^Department of Botany, Faculty of Science, Tanta University, Tanta, Egypt; ^12^Department of Biology, College of Science, United Arab Emirates University, Al-Ain, United Arab Emirates; ^13^Khalifa Center for Genetic Engineering and Biotechnology, United Arab Emirates University, Al-Ain, United Arab Emirates; ^14^Harry Butler Institute, Murdoch University, Murdoch, WA, Australia; ^15^College of Agriculture, Nanjing Agricultural University, Nanjing, China; ^16^Department of Botany and Microbiology, Faculty of Science, Assiut University, Assiut, Egypt

**Keywords:** jasmonic acid, Na^+^ transporter-related gene expression, nutrient homeostasis, salinity, wheat

## Abstract

Salinity is a global conundrum that negatively affects various biometrics of agricultural crops. Jasmonic acid (JA) is a phytohormone that reinforces multilayered defense strategies against abiotic stress, including salinity. This study investigated the effect of JA (60 μM) on two wheat cultivars, namely ZM9 and YM25, exposed to NaCl (14.50 dSm^−1^) during two consecutive growing seasons. Morphologically, plants primed with JA enhanced the vegetative growth and yield components. The improvement of growth by JA priming is associated with increased photosynthetic pigments, stomatal conductance, intercellular CO_2_, maximal photosystem II efficiency, and transpiration rate of the stressed plants. Furthermore, wheat cultivars primed with JA showed a reduction in the swelling of the chloroplast, recovery of the disintegrated thylakoids grana, and increased plastoglobuli numbers compared to saline-treated plants. JA prevented dehydration of leaves by increasing relative water content and water use efficiency via reducing water and osmotic potential using proline as an osmoticum. There was a reduction in sodium (Na^+^) and increased potassium (K^+^) contents, indicating a significant role of JA priming in ionic homeostasis, which was associated with induction of the transporters, *viz., SOS1, NHX2*, and *HVP1*. Exogenously applied JA mitigated the inhibitory effect of salt stress in plants by increasing the endogenous levels of cytokinins and indole acetic acid, and reducing the abscisic acid (ABA) contents. In addition, the oxidative stress caused by increasing hydrogen peroxide in salt-stressed plants was restrained by JA, which was associated with increased α-tocopherol, phenolics, and flavonoids levels and triggered the activities of superoxide dismutase and ascorbate peroxidase activity. This increase in phenolics and flavonoids could be explained by the induction of phenylalanine ammonia-lyase activity. The results suggest that JA plays a key role at the morphological, biochemical, and genetic levels of stressed and non-stressed wheat plants which is reflected in yield attributes. Hierarchical cluster analysis and principal component analyses showed that salt sensitivity was associated with the increments of Na^+^, hydrogen peroxide, and ABA contents. The regulatory role of JA under salinity stress was interlinked with increased JA level which consequentially improved ion transporting, osmoregulation, and antioxidant defense.

## Introduction

Wheat (*Triticum aestivum* L.) is an important cereal crop widely consumed globally as a valuable food. With a dramatically increasing in the human population worldwide and to meet the food demands, wheat production should increase by more than 60% to feed the 9.6 billion world population by 2050 (Tadesse et al., 2019). The major constraints in wheat productivity are abiotic stress such as water stress, heat, and salinity. Although wheat is reported as a moderately salt-tolerant crop, the grain yield could be reduced when the salinity is higher than 10 dSm^−1^ (Munns et al., 2006). Therefore, enhancing wheat yield is the ultimate goal of coping with the food security challenges under changing climate conditions and limited resources.

Salt stress is one of the major environmental issues encountered by plants. It has been determined that about 20% (50 million hectares) of agricultural land is affected by salt stress (Shrivastava and Kumar, 2015). Salt stress impacts the plants' osmotic potential that affects the hydraulic conductivity through reducting water uptake or affecting solute potential (Munns and Tester, 2008). The prolonged exposure and high concentrations of salt ions by plants disturb the Na^+^/K^+^/Ca^++^ homeostasis, increase the release of reactive oxygen species (ROS), and the oxidation of membrane lipids (Ahanger et al., 2017; Sehar et al., 2019). This could cause injuries to the cellular structure of chloroplast and mitochondria, ionic imbalance, and impairment in water relations and photosynthesis, thereby inhibiting plant growth and causing cell death (Sheteiwy et al., 2018; Sehar et al., 2019).

To handle the excessive accumulation of ROS and induced oxidative damages, plant cells intricately synthesize enzymatic antioxidants such as superoxide dismutase (SOD), catalase (CAT), and ascorbate peroxidase (APX) to scavenge ROS (He et al., 2017; Ulhassan et al., 2019; Sheteiwy et al., 2021b). The increased concentration of non-enzymatic antioxidants such as osmoprotectants including proline (Ahmad et al., 2016), non-enzymatic antioxidants (tocopherols), secondary metabolites such as total phenols and flavonoids (Mir et al., 2018), and modulation of phytohormones such as abscisic acid (ABA) and ethylene (Garcia de la Garma et al., 2015) also contribute to alleviating salt stress. At the cellular level, tolerant genotypes use different mechanisms to adapt to salt stress by reducing Na^+^ accumulation in the cytoplasm and restricting its entry into the cell via mediating Na^+^ extrusion using *SOS1* (salt overly sensitive1) and Na^+^ compartmentalization in the vacuole (Shi et al., 2003).

However, tissue tolerance via Na^+^ sequestration in shoots is considered one of the essential mechanisms of salt stress tolerance (Munns et al., 2016). Another salt tolerance mechanism is Na^+^ exclusion from the cytosol by mediating Na^+^ uptake and movement in different organs and/or by Na^+^ compartmentation into the vacuole. Many transporters, such as the high-affinity K^+^ transporters (*HKT1*), plasma membrane Na^+^/H^+^ exchanger (*SOS1, SOS2*, and *SOS3*), H^+^-pyrophosphatase (*HVP*), and tonoplast Na^+^/H^+^ antiporters (*NHX*) have been reported to have an important role in Na^+^ uptake mechanisms and transport pathway (Tuteja, 2007; Shavrukov, 2014). Hence, it is greatly essential to find a practical approach to increase wheat tolerance against stress conditions.

Seed priming is being used to enhance seed germination and crop yield under different environmental stresses (Sheteiwy et al., 2018, 2019; Gao et al., 2020b; Dawood et al., 2021a, 2022; Basit et al., 2022). During seed priming, seed metabolism activation is controlled by the hydration level of seeds, resulting in carbohydrates hydrolysis to be easily taken by the embryo. Seed germination can improve the metabolic process (pre-germinated) to stimulate radicle protrusion (Paparella et al., 2015). During imbibition, seed priming limits the endosperm resistance, nurtures the immature embryos, and repairs membranes (Balestrazzi et al., 2011). In this way, priming can enhance abiotic stress tolerance during dehydration or soaking steps. Seed priming with phytohormones such as jasmonic acid (JA) has been widely utilized to mitigate abiotic stress effects and regulate physio-biochemical processes in plants (Sheteiwy et al., 2018, 2021a).

Earlier studies have reported that seed priming with low concentrations of JA can alleviate salt stress by improving water potential (*via* increased synthesis of osmoregulators), reducing Na^+^ accumulation in tissues, and promoting photosynthesis, growth, and yield of several crops (Sheteiwy et al., 2018, 2021a). For example, it has been reported that seed priming with JA can improve the germination rate, seed vigor, photosynthetic machinery, water use efficiency (WUE), relative water contents (RWC), and metabolites accumulation in soybean (Sheteiwy et al., 2021a), and rice cultivars (Sheteiwy et al., 2018). Similarly, Al-harthi et al. (2021) have found that seed priming with JA can alleviate salt stress in summer squash (*Cucurbita pepo* L.) by upregulating the antioxidant (enzymatic and non-enzymatic) defense system, ionic homeostasis, and pigmentation resulted in higher biomass accumulation.

Hence, the purpose of the current study is to investigate the potential roles of seed priming with JA in adapting the physiological, cellular, and molecular mechanisms of wheat plants exposed to high salt stress. We hypothesize that the primed JA may modulate the osmoregulators, induce the nutrients balance, produce endogenous phytohormones, accumulate secondary metabolites, and stimulate antioxidant defense machinery or stress-related transcripts of wheat plants to improve growth, biomass accumulation, and photosynthetic apparatus. Thus, seeds primed with JA may minimize the effect of salt stress by scavenging the ROS accumulation and improving wheat growth in saline stress conditions.

## Materials and methods

### Experimental design, plant growth, and stress treatment

Field experiments were performed during the 2018/2019 and 2019/2020 growing seasons from November to June at the experimental farm of the Academy of Agricultural Science (JAAS), Nanjing, Jiangsu, China (35.31°N 113.87 °E). The seeds of two cultivars of spring wheat i.e. Zheng Mai9 (ZM9, salt-sensitive) and Yang Mai25 (YM25, salt-tolerant), were brought from the Seed Center of Nanjing, Jiangsu, China (JAAS).

Both cultivars selected in the current experiment were widely cultivated in the delta of the Yangtz river of China, based on their excellent traits, e.g., ZM9 with more resistance to wheat diseases such as *Fusarium* head blight and powdery mildew, YM25 with stronger resistance to lodging, and both showing higher yield and better grain quality. The area has a humid subtropical climate, and the mean temperature and humidity of both growing seasons are shown in Supplementary Table S1. The soil's main properties were as follows: 24.9% clay; 55.6% silt; 19.5% sand. The soil chemical properties from 0 to 30 cm of the experimental site were represented in Supplementary Table S2. The plots' size used in this study was 13 m^2^ (3.25 × 4 m), with a distance between each ridge of 45 cm. The experiments were performed using a complete randomized block design (CRBD, factorial), with three biological replications and eight treatments. The experiment field differs in salinity levels. The plots with 4.6 dSm^−1^ were served as a control, and plots with 14.50 dSm^−1^ were used as salinity stress. This salinity was designed as high salt stress for wheat plants as it was comparable to saline-alkaline lands in the coastal areas of Jiangsu Province, China (Ju et al., 2021). Salt soil plots were established by mixing normal soil with salt (1:100). In order to confine the lateral diffusion of salt with water movement among plots, a plastic membrane was mulched on the ridges of each plot.

For the priming treatments, two levels of JA (0 and 60 μM) were used. Prior to seed priming, diluted sodium hypochlorite was used as a sanitizer solution of wheat grains for 15 min and then washed several times to remove any debrides of the disinfectant. Then, the grains were divided into two sets, a group soaked in distilled water representing the control, and the second group was soaked in JA with a concentration of 60 μM at 15 °C in darkness for 24 h (Sheteiwy et al., 2021a). The time and the selected dose of JA were preliminarily tested and optimized. The seeds were left to dry, and the sowing was performed in the second week of November during the seasons (2018 and 2019).

The seeds were sown in rows at depth of 4 cm and 20 cm space between rows. The recommended amount of essential fertilizers such as N (388 kg h^−1^), P (87.75 kg h^−1^), and K (120 kg h^−1^) was applied according to the soil fertility status. The entire P and K fertilizers were provided before planting as basal doses; however, the doses of N were completely applied at the stem elongation and node formation stages (after 50 days from sowing). The water requirement was supplied to soils based on the wheat growth stages, in which 15, 25, 30, and 25% were supplied to the soil at the initial stage, vegetative growth, reproductive stage, grain filling stage, respectively of the total water requirements. In comparison, the soil was naturally dried during maturation (5%). Irrigation water was supplied and pumped to the plots from a pond through pipes. The irrigation system has pipes buried in the soil at a specific depth and water is pumped to enter these pipes for irrigation. This system was equipped with a water meter to control the water flow to the plots at the different growth stages. Other agricultural practices for wheat production were similar to those applied by farmers' practices in the Jiangsu region of China.

In order to determine the physiological, morphological, and yield, 20 wheat plants with the same vigor were selected, tagged in each plot, and used for the different analyses. The morphological and physiological parameters were recorded after 60 days after sowing. Plant heights were measured from the base to the tip of the plant shoot. The fresh weight (FW) and dry weight (DW; dried in an oven at 80 °C for 24 h) of treated plants were recorded. After the maturity of plants and spikes, the spike/m^2^, grains/ spike, and weight of 100 grains were estimated and it was determined during only the first growing season (2018/2019).

### Determination of photosynthetic pigments, photosynthesis efficiency, and transpiration

The leaf physiological traits such as stomatal conductance (g_s_), transpiration rate (T_r_), net photosynthetic rate (P_n_), and intercellular CO_2_ (C_i_) were determined using a portable photosynthesis system (LI-6400; Lincoln, NE, USA) after 2 h of acclimation in the growth cabinet at 27 °C, 70% relative humidity, the light intensity of 1000 μ mol m^−2^ s^−1^, and CO_2_ of 380 μ mol mol^−1^ (Sheteiwy et al., 2021a). The chlorophyll contents were measured spectrophotometrically using the method described previously by Sheteiwy et al. (2018). Briefly, the fresh leaf samples (0.2 g) were ground in 3 mL ethanol (95%, v/v). The homogenate was centrifuged at 5,000 x*g* for 10 min and the supernatant was extracted. The mixture was then determined by monitoring the absorbance at the wavelengths 665, 649, and 470 nm using a spectrophotometer (UV-2101/3101 PC; Shimadzu Corporation, Analytical Instruments Division, Kyoto, Japan). The chlorophyll fluorescence (CF) was measured by the chlorophyll fluorometer (IMAG-MAXI, Heinz Walz, Effeltrich, Germany) in the expanded leaves after dark adaptation for 15 min according to the methodology of Ali et al. (2015).

### Ultrastructural changes in wheat leaves

To deeply observe the changes in the cellular structures of the plant cell and major structural organelles in terms of chloroplast, cell wall, and starch grain and their functions in response to salt stress and JA priming, the leaf ultrastructural changes were investigated using a protocol reported by Yang et al. (2021) with slight modifications. Segments (6–8 per treatment) were randomly selected from wheat plants and overnight immersed in glutaraldehyde (2.5%, v/v) and washed four times with.1 M of phosphate-buffered saline (PBS), with 10-min intervals between each washing. Then, at 15–20 min intervals, the samples were dehydrated in a graded series of ethanol (50%, 60%, 70%, 80%, 90%, 95%, and 100%) and then washed with absolute acetone for 20 min. The samples were then assembled and fixed on copper grids to be scanned using the transmission electron microscope (JEOL TEM-1230 EX), as described by Yang et al. (2021).

### Determination of Na^+^ accumulation and k^+^ uptake in the different tissues of wheat

The Na^+^ and K^+^ contents of the blade, sheath, stem, and roots of both wheat cultivars were recorded using the method described by Zhao et al. (2014). The tissues were washed with distilled water and then dried at 50 °C for 4 d. After that, the dried samples were ground into a fine powder by liquid nitrogen and digested in nitric acid (5 mL) overnight. Then, Na^+^ and K^+^ contents of different tissues were monitored using a flame photometer following the methodology of Zhao et al. (2014).

### Determination of water-relation parameters

The water relation parameters such as water potential and osmotic potential were measured following the protocol reported by Ahmed et al. (2013). Briefly, the segments of the fully expanded leafy sample were macerated in a mortar. After extract filtration, the sap was centrifuged at 10,000 x g for 10 min at 4 °C, then the supernatant was used to estimate osmolality (c) using a vapor pressure osmometer (Wescor Inc., Logan, UT, USA). WUE was measured as the ratio between P_n_ and T_r_ as described by Jones (2013). Briefly, fresh eight of the leaf sample, and the dry weight (for 48 h in an oven at 75 °C) were recorded and used for the RWC determination. The leaf was then soaked for 24 h in distilled H_2_O. The leaves were then dried using tissue paper prior to turgid weight calculation and the RWC was measured. The RWC was quantified in tissues using the equations of Barr and Weatherley (1962) as follows:


$$RWC\;(\%)\;=\frac{Fresh\;weight-Dry\;weight}{Turgid\;weight-Dry\;weight}\;\times \;100$$

### Determination of H_2_O_2_, proline, phenolic metabolism, and antioxidants profile

The hydrogen peroxide (H_2_O_2_) content was measured in the root and shoot according to Halliwell et al. (1987). Briefly, the root and shoot samples (0.5 g) were macerated in trichloroacetic acid (5.0 mL of 0.1%), and the supernatant was mixed with 1 mL of 1 mM KI, and the absorbance at the wavelengths 390 nm by using spectrophotometer was monitored. Proline was determined in the root and shoot samples according to Sheteiwy et al. (2021b). The leaf samples (100 mg) were homogenized in 3% sulfosalicylic acid (5 mL) and centrifuged at 5,000 x*g* for 10 min. The supernatant was mixed with acid-ninhydrin and acetic acid and boiled for 1 h at 100 °C followed by incubation in an ice bath. The absorbance of chromophore-containing toluene was monitored at 520 nm. Tocopherol in root and shoot samples was recorded at 520 nm following Backer et al. (1980). The flavonoid content in root and shoot samples was determined according to Zhishen et al. (1999). Total phenolic content in root and shoot were quantified and calculated using gallic acid (Sigma-Aldrich Chemie GmbH, Taufkirchen, Germany) as a standard (Shohag et al., 2012).

The antioxidant enzyme activities, such as APX, SOD, and Phenylalanine ammonia-lyase (PAL) were determined in the leaf and root tissues spectrophotometrically at 290 nm by the method of Sheteiwy et al. (2021a). Briefly, fresh leaf and root samples (0.5 g) were homogenized in liquid nitrogen and then macerated in K-phosphate buffer (8 mL, 50 mmol L^−1^, pH 7.8) in ice-cold conditions. The extract was then centrifuged at 10,000 x *g* for 20 min at 4 °C where the supernatant was utilized for the aforementioned enzyme activity measurements.

APX activity was determined following the methodology of Nakano and Asada (1981). The reaction mixture was composed of 1.4 mL PBS (25 mmol L^−1^ pH 7.0 + 2 mmol L^−1^ ethylene diamine tetra acetic acid (EDTA), 300 mmol L^−1^ H_2_O_2_, 7.5 mmol L^−1^ ascorbic acid 100 μL, and 100 μL enzyme extract in a total volume of 1.7 mL. APX enzyme was measured by noticing the reduction percentage of the absorbance of ascorbic acid at 290 nm for 2 min. SOD activity was measured according to the method of Giannopolitis and Ries (1977). The reaction mixture contained 3 mL 50 mmol L^−1^ phosphate buffer (pH 7.8), 13 mmol L^−1^ methionine, 1.22 mmol L^−1^ riboflavin, 78.2 μmol L^−1^ EDTA, 56 μmol L^−1^ of Nitro blue tetrazolium (NBT) and 100 μL extract. The mixture was illuminated and the reaction was used to measure the inhibition of the photochemical reduction of NBT at 560 nm. One unit of SOD was defined as the amount required to inhibit the photoreduction of NBT by 50%. SOD activity was expressed as mg^−1^ protein as reported earlier by Zhu et al. (2015).

### Determination of hormones profile

The concentrations of cytokinins (CKs) were determined according to the method of Rauf and Sadaqat (2007). The indole acetic acid (IAA) was measured following the methodology of Sheteiwy et al. (2021c). Abscisic acid (ABA) was quantified in shoot and root tissues following the methodology of Gao et al. (2020a). For JA determination, frozen root and shoot samples (200 mg) were ground into fine powder using liquid nitrogen. The extracts were then centrifuged at 12000 x *g* for 10 min at 4 °C, and then JA was quantified using liquid chromatography-mass spectrometry (Waters, Milford, Massachusetts, USA) as described by Tsukahara et al. (2015).

### Determination of relative gene expression level

The transcript levels of Na^+^ uptake and transport genes such as *SOS1-NHX2- HVP1* were analyzed in leaf blade-, leaf sheath, stem, and root tissues. The total RNA isolation was performed using an RNA isolation (Takara Bio Inc., Shiga, Japan). cDNA was synthesized using Primer Script RT reagent Kit (Takara Bio Inc.) from 1 μ g of total RNA in a 20 μ L reaction, and diluted 4-fold with water. SYBR premix EX Taq (Takara Bio Inc.) was used to perform the quantitative real-time (qRT-PCR) analysis. The frozen tissues (100 mg) were homogenized in liquid nitrogen and qRT-PCR was performed following the methodology of Gao et al. (2020b) using primers used previously by Darko et al. (2017). ACT1 was used as a control to determine the transcription level of the other studied genes. The PCR program was the same as followed by Salah et al. (2015).

### Statistical analysis

Two-way analysis of variance (ANOVA) was applied to determine differences among treatments. The results were described as the means of three replicates ± SD. The data were analyzed using the statistical package (IBM-SPSS, 19, USA). Mean values were compared by applying Duncan's multiple range test at the.05 level of significance. Asterisks indicate significant differences: ^*^P < 0.05, ^**^P < 0.001, ^***^P < 0.0001. The Hierarchical Cluster Analysis (HCA) and Principle Component Analysis (PCA) were performed through the SPSS program on log_10_ of the obtained data.

## Results

### Effect of JA priming on wheat growth under salt stress

Results revealed that salt stress caused a significant (*P* ≤ 0.05) reduction in growth and numbers of tillers in wheat seedlings compared to the control seedlings (Table 1). The reduction of shoot length, FW, and DW in response to salinity reached 23, 40, and 44% in the ZM9 cultivar and 22, 45, and 44% in cultivar YM25, respectively, compared with the control. Priming with JA resulted in improved plant height, FW, and DW as well as tiller numbers in both wheat cultivars during both growing seasons, compared to unprimed plants (Table 1).

**Table 1:** Effects of JA priming on plant height, FW, DW, number of tillers/plants, T_r_ and P_n_ of two wheat cultivars Mai9 (ZM9, salt-sensitive) and Yang Mai25 (YM25, salt-tolerant) under control and salinity stress conditions during two consecutive growing seasons 2019 and 2020.

	**Treatments**	**Plant height (cm)**	**FW (g)**	**DW (g)**	**Number of tillers plants^−1^**	**T_r_ (mmol H_2_O m^−2^ s^−1^)**	**P_n_ (μmol CO_2_ m^−2^ s^−1^)**
**2019**							
ZM9	Ck	80.43 ± 2.0c	2.53 ± 0.15b	0.624 ± 0.03b	6.33 ± 0.5b	6.29 ± 0.34bc	3.31 ± 0.38b
	60 μM JA	91.50 ± 3.4b	3.36 ± 0.15a	0.831 ± 0.04a	7.33 ± 0.5a	8.32 ± 0.13a	5.14 ± 0.5a
	S1	62.73 ± 2.4e	1.50 ± 0.10c	0.356 ± 0.03c	4.33 ± 0.5c	3.41 ± 0.20fg	2.20 ± 0.10c
	S1+60 μM JA	71.23 ± 1.3d	2.16 ± 0.15b	0.540 ± 0.03b	5.66 ± 0.5b	5.49 ± 0.31de	3.08 ± 0.12b
YM25	Ck	80.83 ± 1.3c	2.59 ± 0.14b	0.634 ± 0.02b	6.00 ± 0.1b	5.61 ± 0.51c-e	3.43 ± 0.19b
	60 μM JA	96.33 ± 1.7a	3.63 ± 0.15a	0.901 ± 0.03a	7.33 ± 0.5a	7.87 ± 0.32a	5.30 ± 0.39a
	S1	63.83 ± 1.4e	1.43 ± 0.15c	0.348 ± 0.04c	4.00 ± 0.10c	3.60 ± 0.49fg	1.92 ± 0.06c
	S1+60 μM JA	73.73 ± 1.1d	2.20 ± 0.17b	0.533 ± 0.05b	6.00 ± 0.3b	5.73 ± 0.32c-e	3.25 ± 0.17b
**2020**							
ZM9	Ck	79.53 ± 1.7c	2.56 ± 0.3b	0.628 ± 0.08b	6.00 ± 0.3b	6.44 ± 0.5b	3.25 ± 0.17b
	60 μM JA	96.66 ± 2.2a	3.23 ± 0.1a	0.798 ± 0.03a	7.66 ± 0.5a	7.63 ± 0.4a	5.34 ± 0.5a
	S1	63.46 ± 3.0e	1.50 ± 0.10c	0.367 ± 0.02c	4.66 ± 0.5c	3.28 ± 0.24g	2.38 ± 0.6**c**
	S1+60 μM JA	72.36 ± 2.2d	2.20 ± 0.10b	0.526 ± 0.03b	6.00 ± 0.3b	5.10 ± 0.21e	3.21 ± 0.11b
YM25	Ck	80.53 ± 0.9c	2.56 ± 0.15b	0.627 ± 0.02b	6.33 ± 0.5b	5.41 ± 0.50de	3.57 ± 0.16b
	60 μM JA	95.06 ± 1.5ab	3.36 ± 0.68a	0.833 ± 0.16a	7.66 ± 0.5a	7.92 ± 0.30a	5.76 ± 0.9a
	S1	61.36 ± 3.5e	1.53 ± 0.15c	0.377 ± 0.04c	4.33 ± 0.5c	4.07 ± 0.17f	2.06 ± 0.07c
	S1+60 μM JA	73.16 ± 2.9d	2.30 ± 0.10b	0.570 ± 0.01b	6.33 ± 0.5b	5.92 ± 0.5b-d	3.23 ± 0.15b
Year (Y)		Ns	Ns	ns	ns	***	***
Cultivars (C)		**	Ns	ns	ns	**	***
Treat. (T)		***	***	***	***	***	***
Y × C		***	Ns	ns	ns	***	***
Y × T		**	Ns	ns	ns	***	***
C × T		*	Ns	ns	ns	***	***
Y × C × T		**	Ns	ns	ns	***	***

*Means sharing the same letters, for a parameter during a year, do not differ significantly at P ≤ 0.05 among the studied factors after the Student–Newman–Keul test*.

^*^P < 0.05; ^**^P < 0.001; ^***^P < 0.0001.

*FW/DW, fresh/dry weight; T_r_, transpiration rate; P_n_, net photosynthetic rate; Ck, control; S, salt treatment; JA, jasmonic acid*.

Comparing both cultivars, the highest DW and FW values were observed in the YM25 cultivar primed with JA in both seasons. On the other hand, JA-primed plants of YM25 in 2019 and ZM9 in 2020 attained taller plants than the corresponding non-primed plants. We noticed that both cultivars showed a similar pattern of growth upon the treatment with 60 μM JA (Table 1). Plant height, FW, DW, and the numbers of tillers were significantly (*P* ≤ 0.05) enhanced by JA priming under salt stress compared to the salt-stressed plants of both cultivars during the two growing seasons.

### Effect of JA priming on photosynthetic pigment, photosynthesis efficiency, and transpiration rate under salt stress

As presented in Table 1, and compared with the control, P_n_ and T_r_ were enhanced in the salt-stressed primed with JA compared to salt-stressed non-primed plants, which had reductions in both traits. The primed plants of the cultivar ZM9 showed the highest value of Tr in 2019, while the cultivar YM25 produced the highest value of Tr in 2020 growing season (Table 1). A significant decrease was observed in C_i_, g_s_, chlorophyll a (Chl *a*), Chl *b*, total chlorophyll, and Chlorophyll fluorescence (FC) in the salt-stressed plants compared to the control plants (Table 2). Interestingly, C_i_, g_s_, Chl *a*, Chl *b*, carotenoids, and CF [represented by maximal PSII photochemical efficiency (Fv/Fm)] were increased in the JA-primed control compared to the non-primed seedlings (Table 2). This could potentially mitigate the damaging effect of salinity stress in both wheat cultivars; yet, still lower than the control plants in both growing seasons.

**Table 2:** Effects of JA priming on C_i_, g_s_, Chl a and b, carotenoids, and CF (FV/Fm) of two wheat cultivars Mai9 (ZM9, salt-sensitive) and Yang Mai25 (YM25, salt-tolerant) under control and salinity stress conditions during two consecutive growing seasons 2019 and 2020.

	**Treatments**	**C_i_ (μmol CO_2_ mol^−1^)**	**g_s_ (mol H_2_O m^−2^ s^−1^)**	**Chl a (mg g^−1^ DW)**	**Chl b (mg g^−1^ DW)**	**Carotenoids (mg g^−1^ DW)**	**CF (FV Fm^−1^)**
**2019**							
ZM9	Ck	541.0 ± 18cd	0.820 ± 0.03c	0.176 ± 0.04cd	0.151 ± 0.07b	0.131 ± 0.04e-g	0.793 ± 0.05c-e
	60 μM JA	627.33 ± 16ab	1.19 ± 0.04a	0.250 ± 0.20a	0.180 ± 0.02a	0.200 ± 0.01b	0.926 ± 0.02ab
	S1	243.67 ± 13f	0.363 ± 0.05ef	0.143 ± 0.15f	0.120 ± 0.09d	0.114 ± 0.05gh	0.406 ± 0.05gh
	S1+60 μM JA	397.67 ± 47e	0.520 ± 0.11d	0.160 ± 0.01d-f	0.148 ± 0.07bc	0.159 ± 0.06cd	0.756 ± 0.03e
YM25	Control	576.00 ± 70b-d	0.773 ± 0.08c	0.183 ± 0.02cd	0.146 ± 0.08bc	0.144 ± 0.04de	0.833 ± 0.02 b-e
	60 μM JA	618.33 ± 25ab	1.20 ± 0.11a	0.253 ± 0.03a	0.172 ± 0.01a	0.220 ± 0.01a	0.953 ± 0.02 a
	S1	224.33 ± 50f	0.390 ± 0.07d-f	0.150 ± 0.01ef	0.111 ± 0.01d	0.123 ± 0.05f-h	0.476 ± 0.07 fg
	S1+60 μM JA	387.67 ± 11e	0.510 ± 0.02d	0.178 ± 0.07cd	0.138 ± 0.02c	0.165 ± 0.05c	0.816 ± 0.15 b-e
**2020**							
ZM9	Ck	520.67 ± 9d	0.720 ± 0.06c	0.170 ± 0.09c-e	0.149 ± 0.07bc	0.130 ± 0.01e-g	0.776 ± 0.15 de
	60 μM JA	608.33 ± 24a-c	1.07 ± 0.08ab	0.220 ± 0.01b	0.176 ± 0.05a	0.203 ± 0.02ab	0.896 ± 0.01 a-c
	S1	218.33 ± 11f	0.333 ± 0.05f	0.144 ± 0.05f	0.112 ± 0.02d	0.110 ± 0.01h	0.343 ± 0.07 h
	S1+60 μM JA	405.00 ± 18e	0.500 ± 0.10de	0.173 ± 0.05cd	0.154 ± 0.07b	0.160 ± 0.01cd	0.770 ± 0.02 e
YM25	Ck	541.33 ± 71cd	0.713 ± 0.06c	0.188 ± 0.02c	0.143 ± 0.08bc	0.141 ± 0.01d-f	0.886 ± 0.07 a-d
	60 μM JA	661.67 ± 52a	0.980 ± 0.10b	0.240 ± 0.01ab	0.167 ± 0.09a	0.220 ± 0.02a	0.970 ± 0.01 a
	S1	212.67 ± 17f	0.346 ± 0.07f	0.148 ± 0.08ef	0.111 ± 0.01d	0.123 ± 0.05f-h	0.510 ± 0.06f
	S1+60 μM JA	369.67 ± 54e	0.456 ± d-f	0.177 ± 0.06cd	0.141 ± 0.07bc	0.170 ± 0.01c	0.836 ± 0.02b-e
Year (Y)		***	***	*	ns	ns	*
Cultivars (C)		***	Ns	***	***	ns	***
Treat. (T)		***	***	***	***	***	***
Y × C		***	Ns	ns	ns	ns	***
Y × T		***	***	***	*	ns	***
C × T		***	Ns	ns	ns	ns	***
Y × C × T		***	*	*	ns	ns	***

*Means sharing the same letters, for a parameter during a year, do not differ significantly at P ≤ 0.05 among the studied factors after Student—Newman—Keul test*.

^*^P < 0.05; ^**^P < 0.001; ^***^P < 0.0001.

*C_i_, intercellular CO_2_; g_s_, stomatal conductance; Chl, chlorophyll; CF, chlorophyll fluorescence; Ck, control; S, salt treatment; JA, jasmonic acid*.

### JA priming reduced Na^+^ accumulation and improved K^+^ uptake in wheat tissues

In comparison to the control plants (no salinity stress), there were considerable increases in Na^+^ concentrations in different tissues of wheat (blade, sheath, stem, and roots) of the two wheat cultivars in both growing seasons in response to salt stress (Table 3). These cultivars showed the highest accumulation of Na^+^ content in the sheath but the lowest in stems. In salt-stressed plants, Na^+^ accumulation was higher in the blades, followed by sheath, roots, and finally, stem in both cultivars during the two experimental years. On average, the increments of Na^+^ content in blade, sheath, root, and stem, were 184.0, 268.0, 227, and 201% in ZM9 and 172, 172, 96, and 133% in YM25, respectively, compared with their corresponding controls.

**Table 3:** Effects of JA priming on Na^+^ concentration and K^+^ concentration in the leaf blade and sheath, stem, and root of two wheat cultivars Mai9 (ZM9, salt-sensitive) and Yang Mai25 (YM25, salt-tolerant) under control and salinity stress conditions during two consecutive growing seasons 2019 and 2020.

	**Treatments**	**Na**^**+**^ **Concentration (mg g**^**−1**^ **DW)**				**K**^**+**^ **Concentration (mg g**^**−1**^ **DW)**			
		**Blade**	**Sheath**	**Stem**	**Roots**	**Blade**	**Sheath**	**Stem**	**Roots**
**2019**									
ZM9	Ck	3.79 ± 0.01f	3.20 ± 0.05c	3.75 ± 0.05e	5.65 ± 0.14d-f	23.12 ± 0.67b	19.88 ± 0.57bc	25.31 ± 0.93c	18.89 ± 0.57e
	60 μM JA	2.31 ± 0.03g	1.98 ± 0.10d	1.51 ± 0.07g	3.41 ± 0.08g	27.12 ± 1.0a	26.01 ± 0.47a	34.38 ± 1.20ab	23.01 ± 0.58a
	S1	10.24 ± 0.3ab	8.58 ± 0.21a	7.57 ± 0.09a	12.84 ± 0.57a	11.90 ± 0.58d	12.00 ± 0.39d	15.68 ± 0.57g	10.49 ± 0.60h
	S1+60 μM JA	4.79 ± 0.03d	3.36 ± 0.06bc	4.02 ± 0.07cd	6.16 ± 0.12d	19.21 ± 0.94c	18.25 ± 0.05c	23.32 ± 0.69c-f	20.64 ± 0.55bc
YM25	Ck	3.65 ± 0.07f	3.21 ± 0.10bc	3.84 ± 0.10de	5.48 ± 0.12ef	22.78 ± 0.38b	19.44 ± 0.59bc	24.69 ± 0.57cd	19.61 ± 0.33de
	60 μM JA	2.21 ± 0.11g	1.95 ± 0.21d	1.68 ± 0.07fg	3.53 ± 0.08g	26.68 ± 1.0a	26.35 ± 0.96a	35.05 ± 2.09ab	23.01 ± 0.22a
	S1	9.96 ± 0.23bc	8.73 ± 0.25a	7.53 ± 0.37a	12.82 ± 0.6a	11.58 ± 0.56d	13.68 ± 0.50d	15.65 ± 1.57g	11.66 ± 0.51h
	S1+60 μM JA	4.45 ± 0.19e	3.41 ± 0.07bc	4.04 ± 0.09cd	6.17 ± 0.04d	18.96 ± 1.2c	18.96 ± 1.15bc	21.34 ± 1.06f	19.96 ± 0.64cd
**2020**									
ZM9	Ck	3.70 ± 0.04f	3.19 ± 0.01c	3.85 ± 0.09c-e	5.19 ± 0.25f	21.76 ± 1.4b	21.34 ± 0.20b	24.32 ± 1.05c-e	18.33 ± 0.51g
	60 μM JA	2.33 ± 0.04g	2.04 ± 0.21d	1.69 ± 0.09fg	3.36 ± 0.20g	26.72 ± 2.34a	27.03 ± 0.39a	36.02 ± 0.35a	22.78 ± 0.67a
	S1	10.33 ± 0.16a	8.56 ± 0.29a	7.65 ± 0.23a	12.08 ± 0.7b	12.42 ± 0.89d	13.03 ± 0.67d	13.61 ± 0.53gh	11.19 ± 0.28h
	S1+60 μM JA	4.53 ± 0.24de	3.38 ± 0.06bc	4.10 ± 0.11c	6.09 ± 0.11de	18.53 ± 0.67c	18.55 ± 1.27c	22.65 ± 2.18d-f	18.70 ± 0.46fg
YM25	Ck	3.51 ± 0.13f	3.22 ± 0.12bc	3.72 ± 0.12e	5.12 ± 0.23f	21.68 ± 1.35b	20.39 ± 0.87bc	23.58 ± 1.23c-f	19.52 ± 0.54d-f
	60 μM JA	2.17 ± 0.05g	2.08 ± 0.21d	1.79 ± 0.09f	3.29 ± 0.21g	27.10 ± 0.49a	25.41 ± 0.46a	33.31 ± 0.91b	23.22 ± 0.34a
	S1	9.85 ± 0.30b	8.55 ± 0.18a	7.23 ± 0.11b	11.49 ± 0.3c	12.64 ± 0.56d	11.49 ± 0.76d	13.16 ± 1.13h	11.57 ± 0.20h
	S1+60 μM JA	4.39 ± 0.21e	3.52 ± 0.11b	3.92 ± 0.08c-e	5.73 ± 0.45d-f	17.84 ± 0.85c	19.60 ± 0.55bc	22.05 ± 2.97ef	21.12 ± 0.65b
Year (Y)		ns	***	Ns	***	ns	***	***	***
Cultivars (C)		ns	***	***	***	*	***	***	***
Treat. (T)		ns	***	***	***	***	***	***	***
Y × C		ns	ns	***	***	ns	***	***	***
Y × T		ns	***	***	***	***	***	***	***
C × T		ns	***	***	***	ns	***	***	***
Y × C × T		ns	***	***	***	ns	***	***	***

*Means sharing the same letters, for a parameter during a year, do not differ significantly at P ≤ 0.05 among the studied factors after Student—Newman—Keul test*.

^*^P < 0.05; ^**^P < 0.001; ^***^P < 0.0001.

*Na^+^, sodium; K^+^, potassium; Ck, control; S, salt treatment; JA, jasmonic acid*.

Interestingly, JA priming significantly (*P* ≤ 0.05) restricted the uptake of Na^+^, which resulted in reductions of Na^+^ in the tissues of the tested wheat cultivars during the two growing seasons (Table 3). In contrast, K^+^ content was highly retarded by salinity, mainly in blades (Table 3). Thus, the reduction of K^+^ reached 49, 40, 39, and 45% in the blade, sheath, stem, and root tissues, respectively, in the ZM9 cultivar salt-stressed plants compared to control plants of the same genotype. In the YM25 cultivar, K^+^ was reduced by 50, 30, 41, and 47% in the blade, sheath, root, and stem tissues, respectively, compared to the non-stressed control plants. JA retarded the reduction of K^+^ in different organs partially for more affected organs (blade and sheath) and enhanced its content to be higher than the control plants, especially for root (Table 3).

### Effect of JA priming on water-relation, water, and osmotic potential of wheat under salinity stress

**Figure 1:**
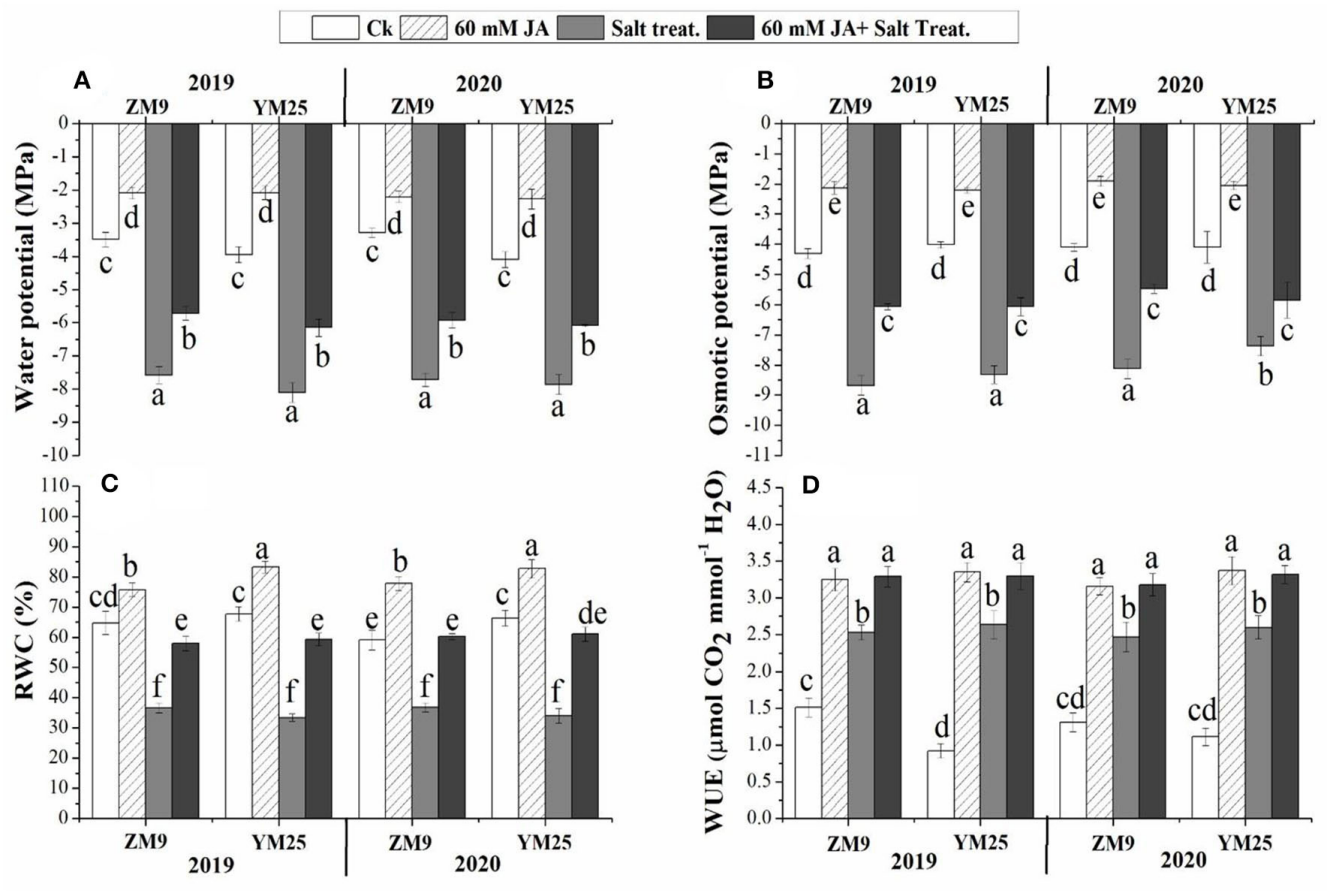
Effects of JA priming on water potential **(A)**, osmotic potential **(B)**, relative water content [RWC, **(C)**], water use effciency [WUE, **(D)**] of two wheat cultivars Mai9 (ZM9, salt-sensitive) and Yang Mai25 (YM25, salt-tolerant) under control and salinity stress conditions during two consecutive growing seasons 2019 and 2020. Means sharing the same letters, for a parameter during a year, do not differ significantly at *P* ≤ 0.05 among the studied factors. Ck, control; S, salt treatment; JA, jasmonic acid.

Plants exposed to salinity stress exhibited significant (*P* ≤ 0.05) reductions in the water and osmotic potential of the studied wheat cultivars during the two growing seasons compared with that in non-salinized control plants (Figures 1A,B). On the other hand, JA priming significantly (*P* ≤ 0.05) increased the water and osmotic potential under saline or non-saline conditions relative to their control. Salinization of soil drastically decreased the RWC of both wheat cultivars comparably throughout the two studied years (Figure 1C). Although WUE was enhanced by salinity, priming in JA profoundly alleviated the damaging impact of salinity on RWC to be statistically highly significant relative to control. These results suggest that JA plays a vital role in alleviating the damage effects of salinity in wheat cultivars through regulating water status and the osmoregulation process.

### Influence of JA priming on H_2_O_2_, proline, secondary metabolites, and antioxidants profile in wheat tissues under salinity stress

**Figure 2:**
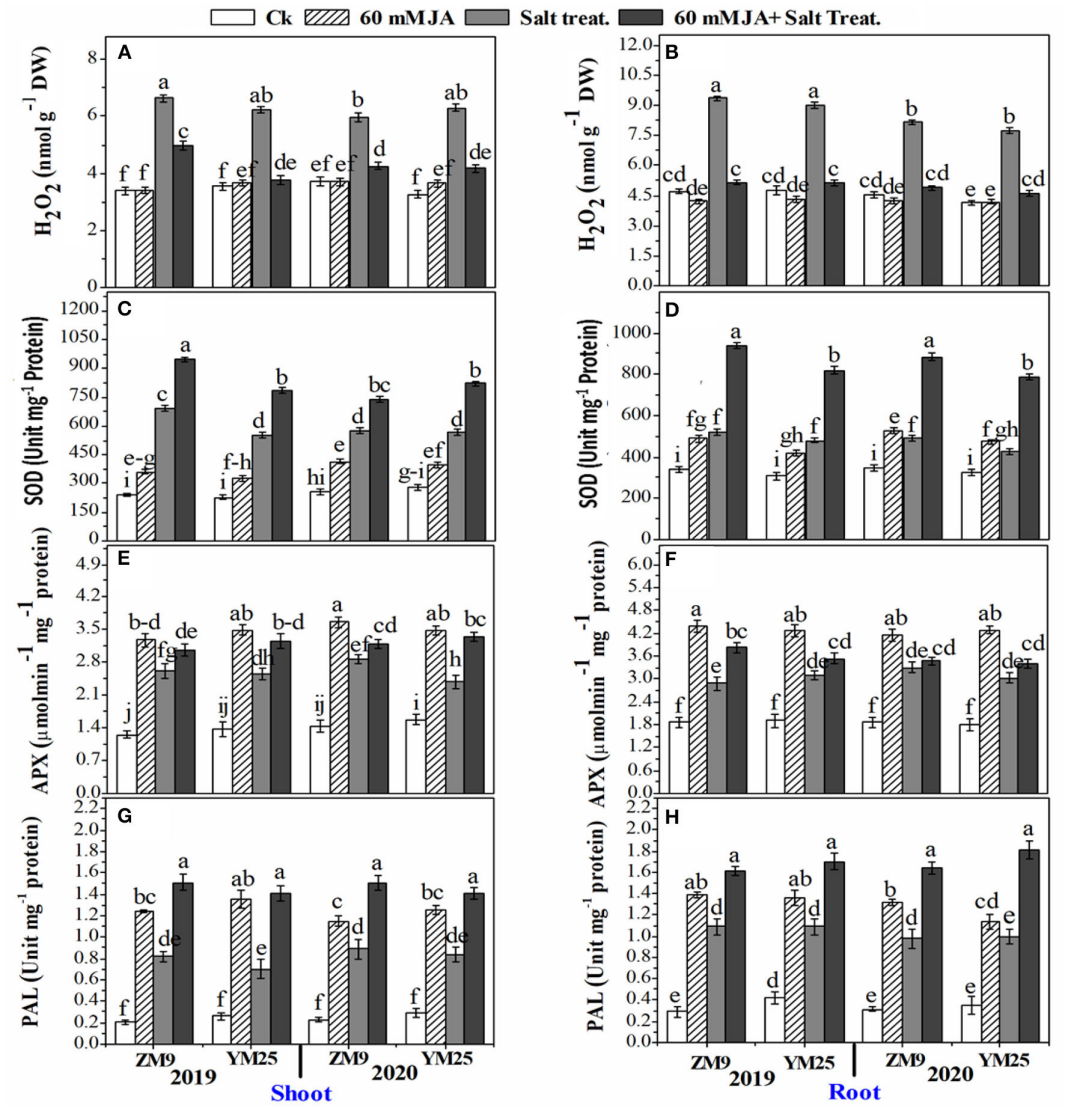
Effects of JA priming on the production of reactive oxygen species (H_2_O_2_) in the shoot **(A)** and root **(B)**; SOD in the shoot **(C)** and root **(D)**; APX in the shoot **(E)** and root **(F)**; PAL in the shoot **(G)** and root **(H)** of two wheat cultivars Mai9 (ZM9, salt-sensitive) and Yang Mai25 (YM25, salt-tolerant) under control and salinity stress conditions during two consecutive growing seasons 2019 and 2020. Means sharing the same letters, for a parameter during a year, do not differ significantly at P ≤ 0.05 among the studied factors. SOD, superoxide dismutase; APX, ascorbate peroxidase; PAL, phenylalanine ammonia-lyase; Ck, control; S, salt treatment; JA, jasmonic acid.

The H_2_O_2_ significantly (*P* ≤ 0.05) increased in response to salinity in the shoots and roots of both cultivars (Figures 2A,B). JA treatment lowered the content of H_2_O_2_ in roots of salt-stressed plants effectively to be comparable to the control plants. In contrast, the effect of JA on the reduction of shoots' H_2_O_2_ was comparable to saline plants which were generally higher than the control (Figure 2A). The specific activities of antioxidant enzymes viz., SOD (Figures 2C,D), and APX (Figures 2E,F) of both wheat cultivars increased by salinity stress in the different tissues in both wheat cultivars. JA profoundly exacerbated the activities of the enzymes in the root and shoot higher than their control.

In non-salinized and salinized plants, JA priming notably enhanced SOD and APX activities compared to the non-JA treated plants in root and shoot tissues of both cultivars (Figures 2E,F). The stimulation power of JA on SOD and APX activities was higher in the salinized plants than in non-salinized ones. PAL activity was also enhanced in response to salinity stress reaching ≥ 6-fold in shoots or roots of the two wheat cultivars throughout the two studied seasons. Further increment of PAL activity was denoted for saline and non-saline plants in plants receiving JA compared to their corresponding control (Figures 2G,H).

**Figure 3:**
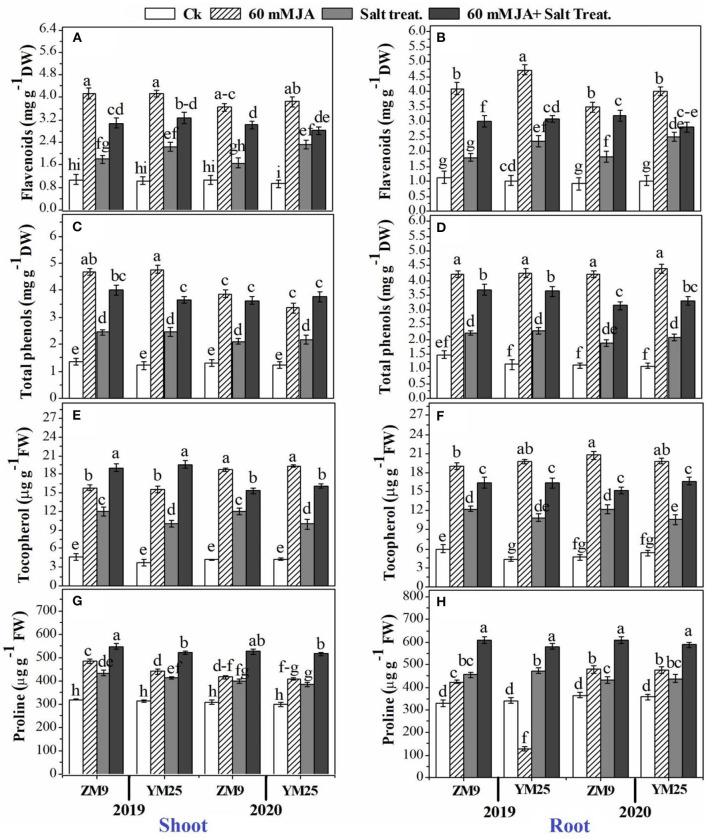
Effects of JA priming on flavonoids in the shoot **(A)** and root **(B)**; total phenols in the shoot **(C)** and root **(D)**; tocopherol in the shoot **(E)** and root **(F)**; proline in the shoot **(G)** and root **(H)** of two wheat cultivars Mai9 (ZM9, salt-sensitive) and Yang Mai25 (YM25, salt-tolerant) under control and salinity stress conditions during two consecutive growing seasons 2019 and 2020. Means sharing the same letters, for a parameter during a year, do not differ significantly at P ≤ 0.05 among the studied factors. Ck, control; S, salt treatment; JA, jasmonic acid.

The contents of proline (an important cell osmolyte) and tocopherols (a non-enzymatic antioxidant) were progressively boosted by salinity stress in the shoots and roots of the two cultivars (Figures 3A–H). The study also included the effect of salinity stress and priming with JA on secondary metabolites of wheat plants. In this regard, flavonoids (Figures 3A,B) and total phenols (Figures 3C,D) accumulated in response to salinity stress in the shoots and roots of both wheat cultivars. JA application had a regulatory role on higher biosynthesis of both metabolites compared to their corresponding control; the highest content was denoted for the non-salinized plants primed with JA. Likely, the antioxidant molecule, tocopherol, was stimulated by soil salinization, and higher exacerbation of tocopherol biosynthesis was also denoted by JA application (Figures 3E,F). Further accumulation of proline could be attributed to the priming of wheat plants with JA. For example, the salinized plants primed with JA demonstrated the highest accumulation capacity of proline in tissues (Figures 3G,H).

### Influence of JA priming on hormonal profile in wheat tissues under salinity stress

**Figure 4:**
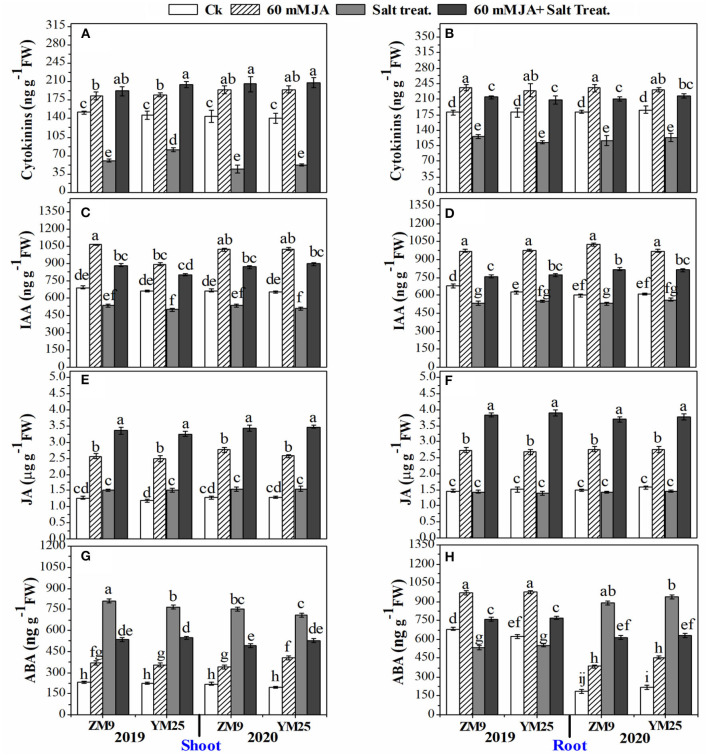
Effects of JA priming on cytokinins in the shoot **(A)** and root **(B)**; IAA in the shoot **(C)** and root **(D)**; JA in the shoot **(E)** and root **(F)**; ABA in the shoot **(G)** and root **(H)** of two wheat cultivars Mai9 (ZM9, salt-sensitive) and Yang Mai25 (YM25, salt-tolerant) under control and salinity stress conditions during two consecutive growing seasons 2019 and 2020. Means sharing the same letters, for a parameter during a year, do not differ significantly at *P* ≤ 0.05 among the studied factors. IAA, indole acetic acid; JA, jasmonic acid; ABA, abscisic acid; Ck, control; S, salt treatment.

The application of JA to wheat seeds caused significant positive changes in hormonal homeostasis (Figure 4). In this regard, the contents of CKs were dramatically (*P* ≤ 0.05) reduced (by 31.9–66.7%) in salt-stressed plants, especially in the shoots, for both cultivars during both growing seasons (Figures 4A,B). However, JA increased the content of CKs in shoots and roots of non-stressed plants compared to the control plants. The JA not only retrieved the contents of CKs in stressed plants higher than their corresponding stressed plants in those not treated with JA but also increased it to higher levels than in the non-stressed plants (Figures 4A,B). Moreover, IAA was highly attenuated in salt-stressed plants with approximately the same effect on both tissues as well as the two tested cultivars during the two seasons with a percent reduction of ~ 23% compared with non-salinized plants (Figures 4C,D).

JA curtailed the reduction of IAA contents in shoots and roots to be higher than in control plants as well as stimulated IAA contents of non-stressed plants, which is recommended for both cultivars, tissues, and studied growing seasons (Figures 4C,D). Except for the stressed shoots of YM25, endogenous JA contents in shoots and roots were not affected by salinity stress shoots and roots (Figures 4E,F). Furthermore, the exogenous application of JA significantly (*P* ≤ 0.05) boosted the JA endogenous content in stressed and non-stressed plants to higher levels than the control treatment (non-primed seeds). It is worth mentioning that the highest endogenous JA contents were noted in JA-primed plants exposed to the high salt concentration.

ABA was differentially affected by salinity stress; the effect depended on the examined tissue and growing season (Figures 4G,H). The shoots of the two cultivars attained significantly (*P* ≤ 0.05) higher levels of ABA under salinity stress regardless of the tested season. However, in the roots, salinity stress reduced ABA contents in 2019 but increased it in 2020 for both cultivars. JA increased ABA contents of shoot tissues in non-stressed plants compared to the corresponding treatment. The levels of ABA in shoots of salinized plants treated with JA were lower than in stressed plants but still higher than in non-stressed plants. ABA contents were significantly (*P* ≤ 0.05) enhanced in the roots of the non-stressed plant primed with JA in the two growing seasons (Figure 4H). On the other hand, JA treatment restrained the reduction of ABA levels in roots in 2019. During the growing season of 2020, the increased levels of ABA were highly (*P* ≤ 0.05) attenuated by salinity stress in response to JA priming, yet the levels were higher than in control plants (Figures 4G,H).

### Effects of JA priming on the relative expression of *SOS1, NHX2*, and *HVP1* in wheat tissues under salinity stress

**Figure 5:**
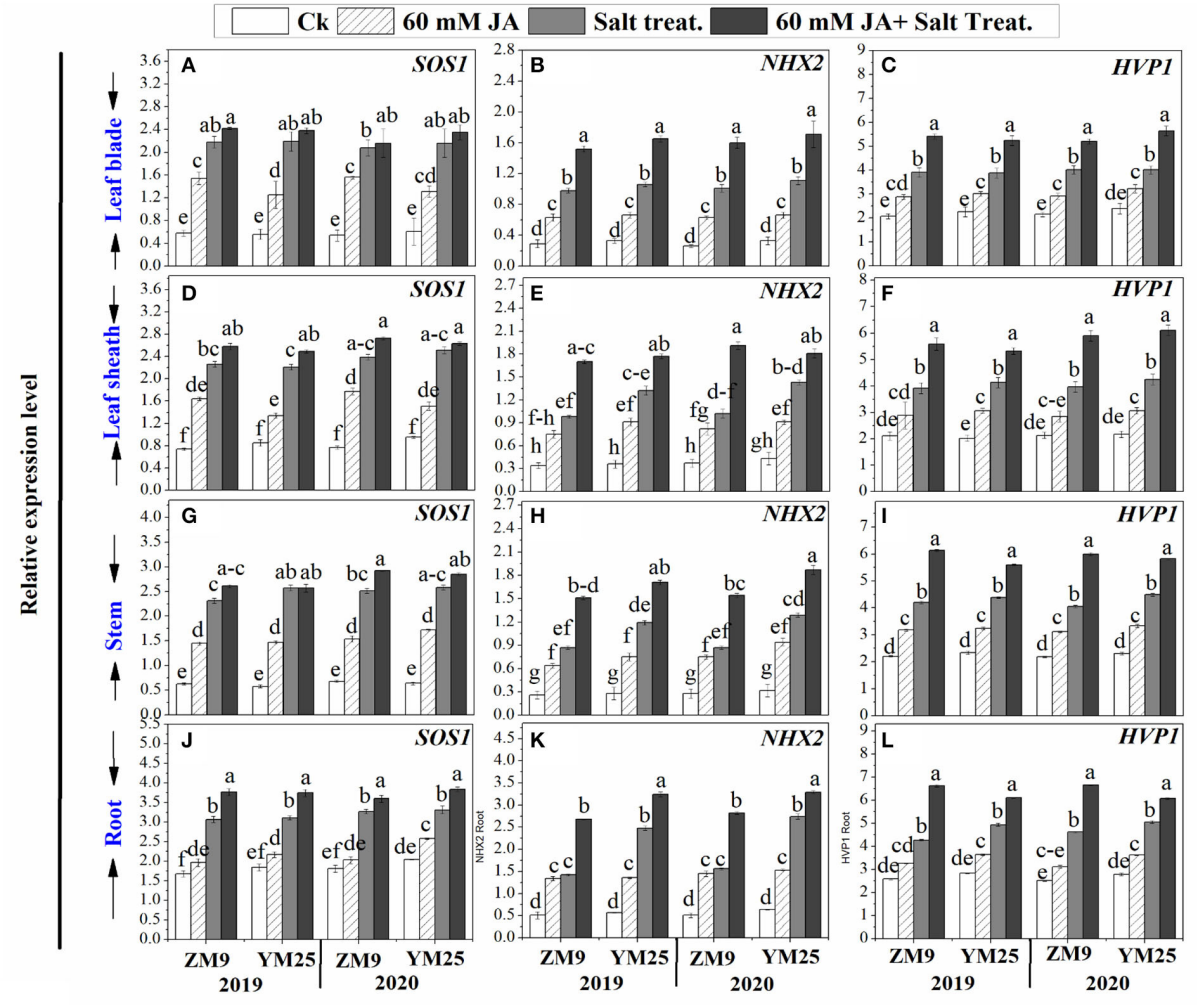
Effects of JA priming on the relative expression of *SOS1, NHX2*, and *HVP1* in leaf blade [**(A–C)** respectively], in leaf sheath [**(D–F)** respectively], in stem [**(G–I)** respectively], and in the root [**(J–L)** respectively] of two wheat cultivars Mai9 (ZM9, salt-sensitive) and Yang Mai25 (YM25, salt-tolerant) under control and salinity stress conditions during two consecutive growing seasons 2019 and 2020. Means sharing the same letters, for a parameter during a year, do not differ significantly at *P* ≤ 0.05 among the studied factors. Ck, control; S, salt treatment; JA, jasmonic acid.

Salinity stress differentially induced the expression levels of *SOS1* based on the organ tested (Figure 5). Upon exposure to high salt, the transcript levels of *SOS1* were significantly (P ≤ 0.05) increased by 2-, 1.5-, 3.1-, and 4.2-fold in roots, stem, leaf sheaths, and blades, respectively compared to the same tissues of non-stressed plants (Figures 5A,D,G,J). Salinity stress induced the expression level of *NHX2* progressively by 5.8-, 3.3-, 3-, and 3-fold and *HVP1* by 1.6-, 1.92, 1.84-, and 1.92-fold for roots, stems, and leaf sheaths, and blades, respectively compared to non-stressed plants. Priming of JA exhibited stimulatory effects on the expression of *SOS1, NHX2*, and *HVP1* genes in wheat plants under saline and non-saline conditions (Figure 5).

### JA upregulated the ultrastructural changes of chloroplast under salinity stress

**Figure 6:**
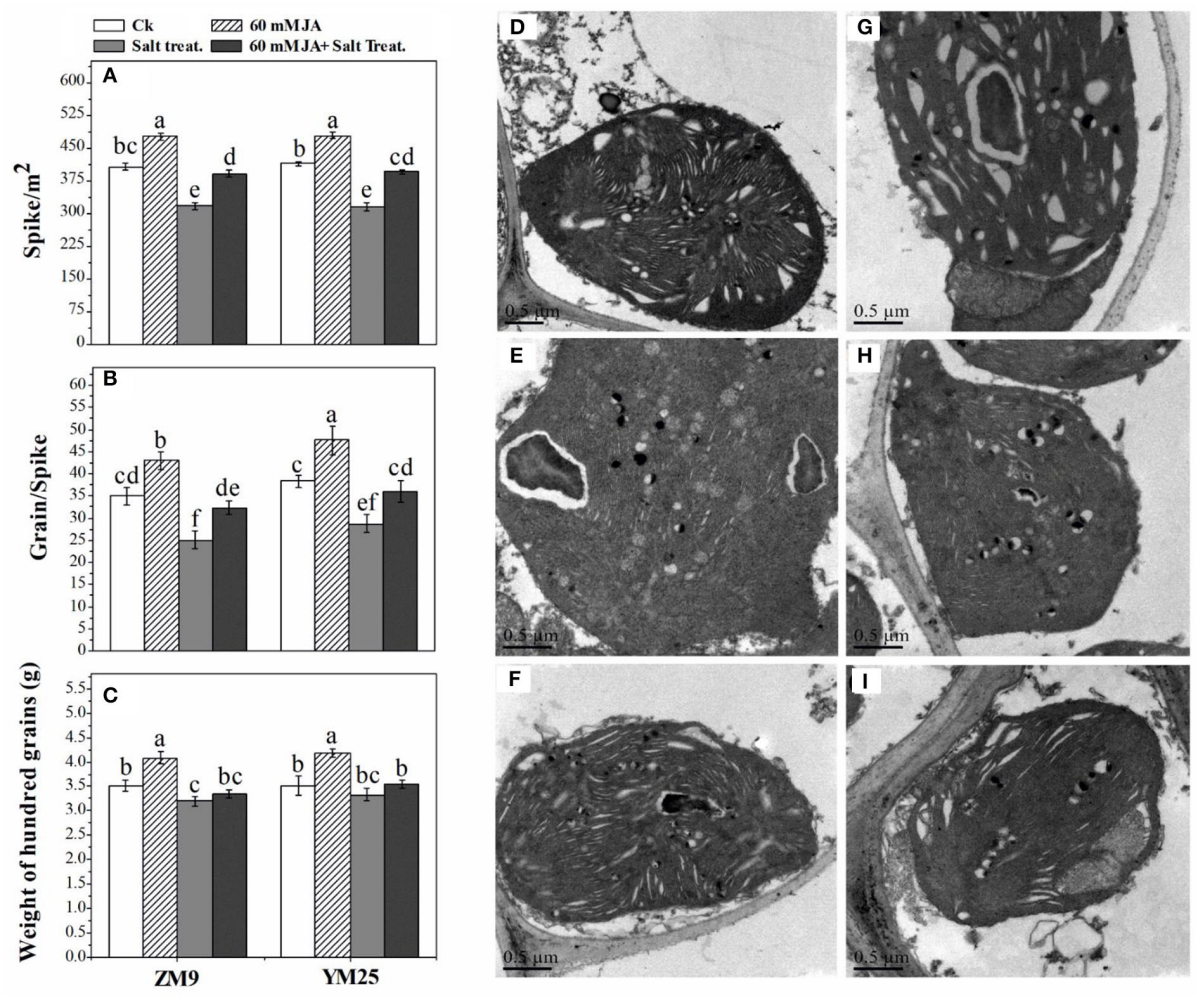
Effect of JA priming on Spike /m^2^ **(A)**, grains/spike **(B)**, and weight of 100 grains [g **(C)**]; the ultrastructure of the leaf of two wheat cultivars Mai9 (ZM9, salt-sensitive) and Yang Mai25 (YM25, salt-tolerant) under salinity stress, CK [without priming under normal conditions in YM25 **(D)** and ZM9 **(G)**]; S1 [exposed to salinity without JA priming in YM25 **(E)** and ZM9 **(H)**]; JA priming+S1 [exposed to salinity with JA priming in YM25 **(F)** and ZM9 **(I)**].

Salinity dramatically affected the ultra-structural configurations of the cell, where the disintegration of plastid compactness in salinized wheat cultivars was the resultant (Figure 6). Due to salinity stress, starch grains and plastoglobuli particles were reduced alongside the loss of the integrity of grana. Interestingly, the cultivar ZM9 exhibited swollen chloroplasts with non-well-developed and sometimes, unrecognizable granum structures compared to its corresponding control or the other wheat cultivar. By combining JA priming and increased NaCl application on plants, the chloroplasts were oval shaped, more consistent with the regular configuration. In addition, several numbers of starch grains and higher plastoglobuli particles relative to deteriorated chloroplasts' of saline treatments were only found in the JA-treated plants (Figure 6).

### Influence of JA priming on yield of wheat cultivars under salinity stress

The number of spike/m^2^ and grains/spikes were dramatically (*P* ≤ 0.05) reduced by 25 and 21%, respectively, in response to salinity stress compared with their relative control (Figures 6A,B). The weight of 100 grains was slightly affected by salinity stress with an 8% reduction in the cultivar ZM9 (Figure 6C). On the other hand, the same stress in the YM25 cultivar did not affect the weight. Intriguingly, JA significantly (*P* ≤ 0.05) increased the yield of non-stressed plants. Salinized-wheat plants treated with JA highly mitigated the detrimental effects of salinity on wheat yield as compared with the unprimed stressed plants.

### Heat map and PCA analyses of traits related to different treatments

**Figure 7:**
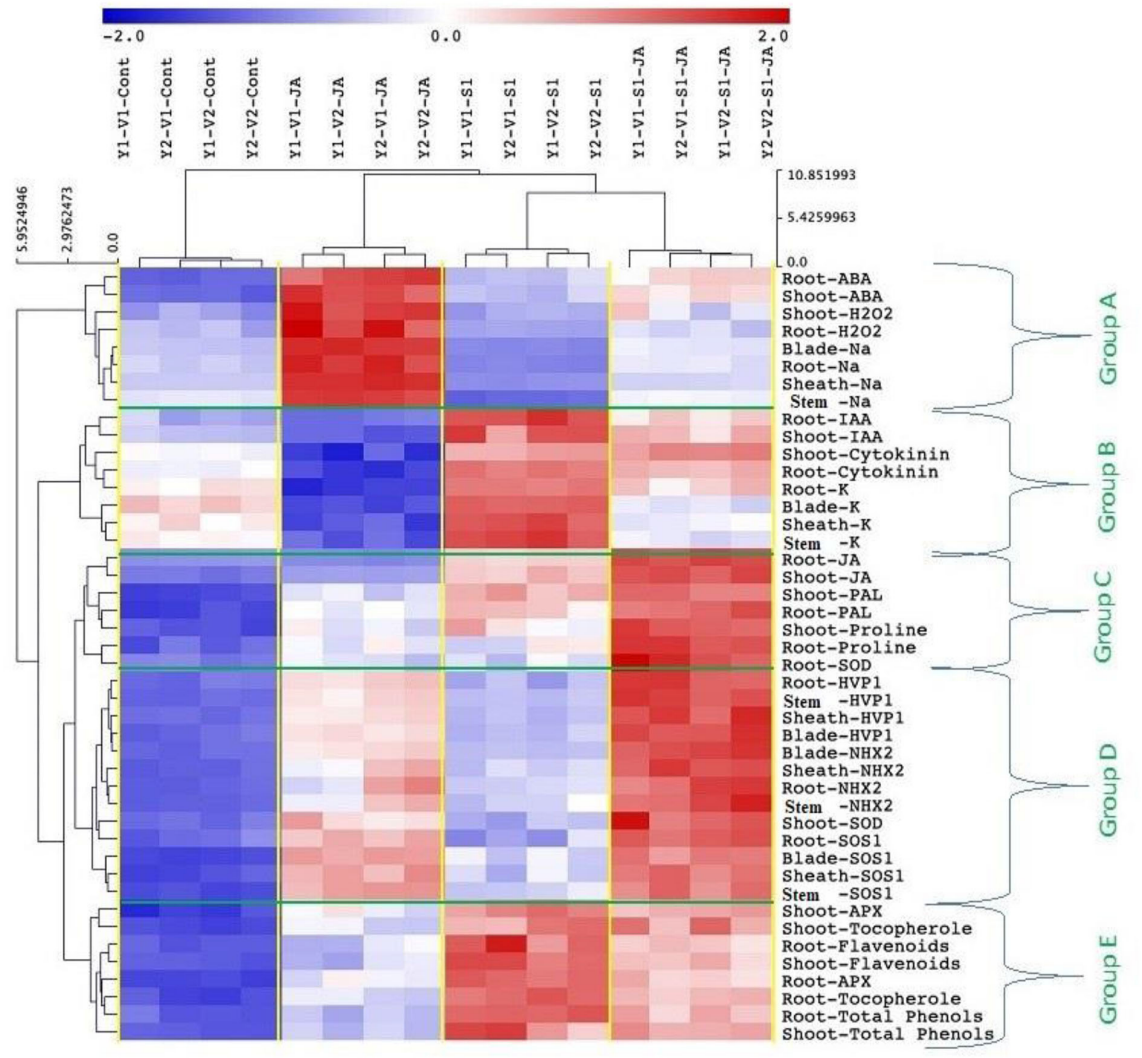
Cluster analysis showing data correlation clustered the metabolic activities to five clusters; Group A) included ABA (shoot and root), H_2_O_2_ (shoot and root), Na^+^ content (root, shoot, blade, and sheath), Group B) included IAA (shoot and root), cytokinins (shoot and root), and K+ (root, stem, blade, and sheath), Group C) included JA (shoot and root), PAL (shoot and root), proline (shoot and root), and SOD activity of roots, Group D) included the expression of genes *HVP1, NHX2*, and *SOS1* of stem, root, leaf sheath, and leaf blade, as well as the SOD activity in the shoot, and Group E) included APX activity (shoot and root), tocopherol (shoot and root), flavonoids (shoot and root), and total phenol (shoot and root). ABA, abscisic acid; IAA, indole acetic acid; JA, jasmonic acid; PAL, phenylalanine ammonia-lyase; SOD, superoxide dismutase; APX, ascorbate peroxidase.

All mean values of the morphological and biochemical parameters were subjected to hierarchical clustering as a heat map of five 5 clusters: Group (A) included ABA and H_2_O_2_ (in shoot and root), and Na^+^ content (in the root, stem, blade, and sheath); Group (B) included IAA and CKs (in shoot and root), and K^+^ (root, shoot, blade, and sheath); Group (C) included JA, PAL and proline (in shoot and root), and SOD activity (in roots); Group (D) included the expression of genes *HVP1, NHX2*, and *SOS1* (in the stem, root, and leaf sheath and blade) and SOD activity (in the shoot); while Group (E) included APX activity, tocopherol, flavonoids and total phenol (in shoot and root) (Figure 7).

**Figure 8:**
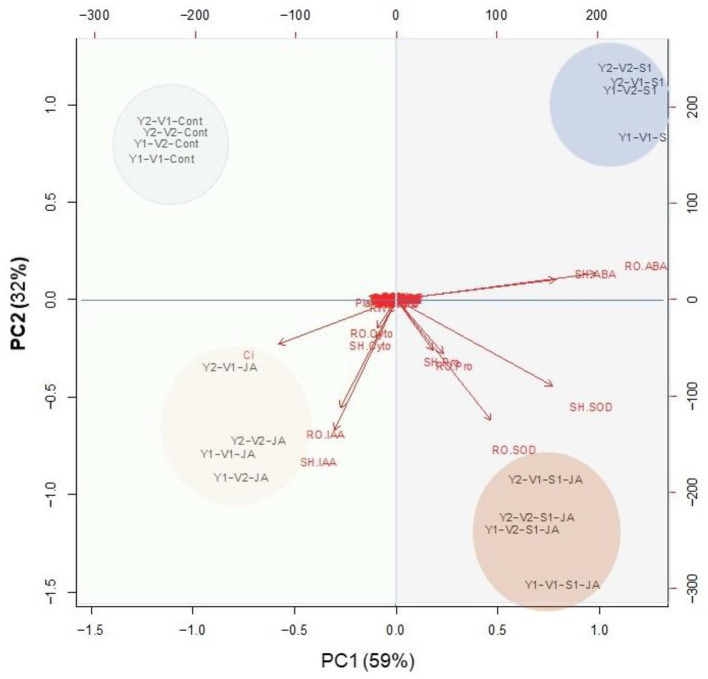
A principal component analysis (PCA) determines the degree of association within the treatments and variables of two wheat cultivars Mai9 (ZM9, salt-sensitive) and Yang Mai25 (YM25, salt-tolerant) primed with jasmonic acid (JA) and grown under salinity stress.

PCA analysis presented in Figure 8 indicated that the variables of group A strongly connected with stressed plants without JA priming, especially ABA. The group–B variables were relatively strongly delineated with JA-primed non-stressed plants (Figure 8) especially Ci, IAA, and cytokinin. On the other hand, the group –C, and –D variables were relatively strongly delineated with JA-primed salinity stressed plants especially proline and SOD (shoot and root).

## Discussion

Many studies found that the endogenous content of JA was boosted under environmental stress. For instance, an enhanced level of endogenous JA due to exposure to salinity stress was demonstrated in many species (Kutik et al., 2004; Zbierzak et al., 2010; Shanmugabalaji et al., 2013; Lohscheider and Bartulos, 2016; Mazur et al., 2020). Thus, higher levels of JA may function as a protection signal against salinity stress in plants (Wang et al., 2020). In the present study, the exogenous application of JA induced multilayered defense mechanisms under salinity stress throughout the two growing seasons. The application of JA significantly increased growth and yields in response to salinity stress, resulting in values close to the control plants without salinity. In general, we found significant differences among treatments (T) for all growth criteria. Except for the plant height, the effect of the year (Y) was not significant, suggesting stability in plant response to JA and/or salinity regardless of the cultivar (C) tested. Similar responses to enhancing growth parameters by JA under salinity stress were previously reported in several species (Yuan et al., 2018; Ali et al., 2019; Liu et al., 2019; Al-harthi et al., 2021; Sheteiwy et al., 2021a).

Notwithstanding, JA stimulated tolerance mechanisms in salt-stressed plants by minimizing Na^+^ absorption and translocation to foliar organs to a level comparable to non-stressed plants. Thus, JA induced the exclusion mechanism for rendering Na^+^ at a lower level under the salinity stress of both wheat cultivars. Reduction of ion toxicity by exogenous JA application could be the main regulatory role for several biochemical pathways in the two wheat cultivars. Shahzad et al. (2015) recorded Na^+^ exclusion by JA in the root by decreasing Na^+^ uptake, mediating salt tolerance in maize genotypes. Na^+^ accumulation detected in different organs of salt-stressed plants is usually associated with a high reduction of K^+^ content, reflecting the antagonistic effect of Na^+^ to K^+^, which is a common metabolic feature under salinity stress (Javed et al., 2021). Thus, the metabolic processes associated with K^+^, such as g_s_, WUE, and photosynthetic efficiency, are retarded in saline environments (Al-harthi et al., 2021). In the current study, there were significant differences in the acquisition of Na^+^ or K^+^ in plant organs, except for blades, among cultivars, treatments, or their interaction. The highly significant Y × C × T interaction indicates the varying responses of genotypes to JA and salinity in each season. The exogenous application of JA on salt-stressed plants mainly retained Na^+^ and K^+^ homeostasis, diminishing ion toxicity, and its consequences. In conformity, the foliar application of JA to the seedlings of strawberries and summer squash plants increased K^+^ content under salt stress (Faghih et al., 2017; Al-harthi et al., 2021).

Plants instigate complex ion transporters to cope with salt stress. In this work, the relative expression levels of genes involved in Na^+^ uptake, transport, and sequestration (*SOS1, NHX2*, and *HVP1*) were upregulated in the blade, sheath, shoot, and roots of the two wheat cultivars under salt treatment. Salt stress caused the transcript level of *SOS1* to increase to its highest in blades and the lowest in roots, and there was a reverse trend in the case of *NHX2*. Such response was accompanied by the highest increase in the percentage of Na^+^ ions in the blade and the lowest increment of Na^+^ in roots, suggesting that sequestration of Na^+^ into vacuoles may be an important component trait for the salt tolerance mechanism in wheat. This response could be interpreted by the enhanced levels of ABA, which reduce Na^+^ exclusion in roots and activate its translocation and accumulation in leaves as was reported by Cabot et al. (2009). Similarly, Yu et al. (2007) stated that ABA stimulates the expression of salt-tolerant genes, *HVP1* and *HVP10*, for Na^+^/H^+^ antiporter and vacuolar H^+^-pyrophosphatases.

The key role of the *NHX2* gene seems to be Na^+^ sequestration to the vacuole. This suggests that Na^+^ accumulation in the vacuoles in the different organs of salinized plants cannot protect the cytosol against excess Na^+^ as has been reviewed by Darko et al. (2017). The higher level of *HVP1* reported in the salinized plants; herein, could be linked to higher WUE observed in salt-stressed plants (Haq et al., 2019). It has been demonstrated that *HVP1* acts as an influential pump that activates the sequestration of toxic salts to the vacuoles. Interestingly, a plant supplemented with JA exhibited a further increase in *SOS1, HVP1*, and *NHX2* expression levels. These observations indicate that Na^+^ exclusion components are highly activated in salt-stressed plants by JA application. It has been reported that JA can increase salinity tolerance through activating Na^+^ efflux and K^+^ influx in tissues where exclusion of Na^+^ was sufficiently documented (Khan et al., 2020). In harmony, the heatmap and PCA analyses highly recommended the role of JA in a synergistic increase in the expression of *SOS1, NHX2*, and *HVP1* in plant organs in response to salinity. Higher salinity levels increase Na^+^ content in plant tissues, which activates K-uptake via enhanced Na^+^ exclusion. In this regard, Malakar and Chattopadhyay (2021) have reported that when the concentration of salt increases, NHX mediates Na^+^ influx into the cytoplasm, affecting membrane depolarization and triggering K^+^ efflux from the cytoplasm.

On the other hand, JA priming can restrain Na^+^ accumulation, which activates vacuolar K^+^ influx without moving Na^+^. In this context, we suggest the priming role of JA to be through upregulating the NHXs that mediate Na^+^ localization in vacuoles, which in turn modulate Na^+^ toxicity *via* using it as a cheap vacuolar osmoticum aid in osmotic adjustment during salt stress (Shabala, 2013). Malakar and Chattopadhyay (2021) have reported that *NHX1* and *NHX2* genes instigate salinity tolerance through enhancing K^+^ level, K^+^ to Na^+^ ratio, and reducing oxidative damage. In addition, Andrés et al. (2014) have referred to ion exchangers, *NHX1* and *NHX2* mediate K^+^ uptake into vacuoles to regulate cell turgor and stomatal function for higher transpiration rates.

Our results showed salinity-induced damage to the chloroplast, grana lamellar organization, and chloroplast swelling. Such damage was more pronounced in the wheat salt-sensitive cultivar, ZM9. Several studies have reported similar modifications in the lamellar organization (Papadakis et al., 2007), swelling of chloroplast lamellae, and undefined grana structure (Štefanic et al., 2013; Hameed et al., 2021). Furthermore, wheat cultivar YM25 exhibited increases in the numbers of plastoglobuli under salinity stress. Similar to our results, salt stress induction enhanced the numbers of plastoglobuli and the disordering of the chloroplast envelope of cucumber leaves (Shu et al., 2012). The enlargement of plastoglobules reported in our study was associated with a disintegration of the thylakoid structure. Bejaoui et al. (2016) attributed the increase in the number and size of plastoglobuli in salt-treated plants of *Sulla carnosa* to a disturbance of the thylakoid membrane. Thus, salinity stress can prevent the formation of thylakoid lamellae from plastoglobules which reflects great damage to chloroplast and their membranes, resulting in low efficiency of the photosynthetic moiety, and thereby, retarding the growth of wheat plants. However, our results indicated that wheat cultivars primed with JA showed a reduction in the swelling of the chloroplast stroma and recovery of the thylakoids grana compared to only saline-treated plants.

Ali et al. (2018) reported a similar recovery of chloroplast ultrastructure in rapeseed by JA application under cadmium stress. It is worth mentioning that the production of JA is linked to plastoglobules which are recruited with JA precursors and four enzymes involved in its production in chloroplasts (Lundquist et al., 2012, 2013; Van Wijk and Kessler, 2017). Under salinity stress, endogenous JA increments, however, are stimulated parallel to the enrichment of the salinized plants' chloroplast with high plastoglobules number, while further stimulation of endogenous JA under the interactive effect of JA and salinity ascribed more efficient chloroplast ultrastructure with a higher number of plastoglobules. It is worth noting that the enhancement of endogenous JA in salinized plants combined with JA application is the main regulatory defense strategy of JA against salinity stress. Likely, the data of the current study exhibited a reduction in shoots' and roots' CKs, but much more so for shoots, accounting for early senescence, reduced pigment content, and may be affected in plastids ultrastructure.

Thus, the observed changes in the endogenous CK contents clearly indicated the involvement of these hormones in plant stress responses. JA adjusted the CKs biosynthesis in different organs under salinity stress to be higher than control plants parallel to better leaves status and higher photosynthetic machinery. Also, high levels of IAA parallel to improvement of growth in response to JA application of stressed and non-stressed plants could be associated with the regulatory role of JA on hormonal homeostasis and induction of plant growth. So, JA interacts with different kinds of hormones to regulate the growth and development of plants such as auxin, cytokinin, and ABA. These interactions may help to optimize the growth and development of plants under abiotic stress conditions. Salinity inhibited photosynthesis by reducing photosynthetic pigments content, the maximal PSII photochemical efficiency (Fv/Fm), P_n_, g_s_, and C_i_. On the other hand, there was an increase in the photosynthetic efficiency with JA priming correlated with the regulation of g_s_, photosynthetic rate, CO_2_ transport rates, and Chl biosynthesis. The photosynthetic-related traits, particularly Fv/Fm, P_n_, and C_i_ were also affected by Y, T, C, or their corresponding interactions. In general, increasing g_s_ and C_i_ by JA indicates a pivotal feature in fulfilling the requirements of photosynthesis by increasing the photosynthetic rate that ensures a high level of photoassimilates for the growing tissues, enhancing salinity tolerance. Besides, the increased Fv/Fm ratio by JA priming also measures the maximum efficiency of photosystem II (PSII, the quantum efficiency), reflecting enough energy for higher photosynthetic efficiency of wheat cultivars under salinity stress (Maxwell and Johnson, 2000).

Similarly, Ghassemi-Golezani and Hosseinzadeh-Mahootchi (2015) reported enhancements in Chl a and b, and PSII (Fv/Fm) of safflower under the interactive effect of JA combined with saline stress. We argue that the enhancement of Chl *a*, Chl *b*, and carotenoids by JA under salinity stress can also be denoted. Thus, this effect was recommended according to the significant variation among treatments recorded without any effect of Y × C or C × T on the studied parameters.

It has also been proposed that plastoglobules actively cope with abiotic stress via the regulation of plastid development and metabolic processes (Rottet et al., 2015). Our data demonstrated that JA further increased α-tocopherol content in response to salinity stress. This confirms that the pre-treatment of JA plays an important role in preventing chloroplast degradation by enhancing the production of protective antioxidant compounds (e.g., α-tocopherols) which tend to conserve membrane against ROS (Bashandy et al., 2020; Younes et al., 2020) and hinder photoinactivation of PSII (Havaux et al., 2005). In this regard, plastoglobules are a sink of lipophilic antioxidants such as α-tocopherol where main enzymes have been present in their membranes (Wójtowicz et al., 2021). The data of current work found that α-tocopherol content elevated parallel to a higher number of plastoglobules as was reported by Wójtowicz et al. (2021). Thus, JA instigates cellular signaling under salinity stress through mediating α-tocopherol content.

The accumulation of Na^+^ in cells affects the water status and osmotic potential of stressed plants, causing osmotic stress. In this study, the negative impact of salinity on water status was coined from a high reduction of RWC, but not WUE. Salinity stress reduces g_s_ and T_r_ which ultimately decreases WC (Pooja et al., 2019). The salinized wheat cultivars exhibited a reduction in osmotic potential, revealing plants cannot take up enough water as the cells have low turgor, as reported by Soni et al. (2021). Interestingly, the enhancement of WUE under salinity stress in both cultivars could be associated with the reduction of g_s_, C_i_, and T_r_, as illustrated by Sandhu et al. (2019). However, JA priming improved RWC, osmotic potential, and WUE in wheat plants treated with salinity, a trend observed in several other studies (e.g., Yosefi et al., 2018; Taheri et al., 2020; Sheteiwy et al., 2021a; Soni et al., 2021).

Nevertheless, JA treatment was not efficient enough to return the osmotic and water potential of the salinized plants to the level of the control plants. The maintenance of turgor under the interactive effect of salinity and JA could be linked with proline accumulation which acts as osmotica co-opted in salinity stress tolerance (El-Sayed et al., 2014; Salimi et al., 2016; Taheri et al., 2020). Thus, we can conclude that JA induces osmotic adjustment, which reduces the osmotic potential by increasing proline content that increases external osmolality, maintaining water absorption, and various physiological processes associated with water availability, which reduce osmotic stress.

ROS are generally accumulated in plant cells under stress and are commonly documented for salinity. In that sense, salinity stimulates electron flows to molecular oxygen (Dawood et al., 2021b; Sofy et al., 2021), causing the enhancement of different forms of ROS. For example, H_2_O_2_ content is enhanced under salinity stress as a secondary stress factor that negatively affects wheat cultivars, causing an oxidative burst. On the other hand, exogenous JA coupled with NaCl treatment significantly reduced the level of H_2_O_2_ in wheat seedlings. This positive effect can be attributed to the potential ability of JA to enhance antioxidant metabolites (e.g., tocopherols and phenolics) and enzyme activities (SOD and APX). Similarly, previous studies have shown that the application of JA effectively suppressed the toxic effects of oxidative burst by enhancing the ROS-scavenging potential of the antioxidant defense system in stressed plants (Sirhindi et al., 2015; Ahmad et al., 2018; Najafi kakavand et al., 2019; Bali et al., 2020; Lang et al., 2020; Kamran et al., 2021). In addition, the activity of PAL was upregulated in response to salinity stress alone or in combination with JA, resulting in increased phenolic and flavonoid contents, ultimately leading to enhanced tolerance to salinity stress.

The heat map analysis clustered the metabolic activities of wheat plants under the combined effects of salinity and JA into five clusters (Figure 7), indicating that JA modulates the salinity effect in wheat plants through different pathways. For example, JA-treated plants retained Na^+^ and K^+^ homeostasis that reduces ion toxicity, which damages cell membranes, and other cellular compartments. On the other hand, K^+^ is required for several physiological and biochemical processes. Besides, JA helps Na^+^ uptake, transport, and compartmentalize in vacuoles to minimize its adverse effects on the biochemical processes in the cytosol. This was facilitated by the accumulation of ABA and the upregulations of genes such as *SOS1* and *NHX2* that help sequester Na^+^ from roots to the leaf vacuoles. Furthermore, JA priming helps the recovery of chloroplast ultrastructure, increasing the photosynthesis efficiency in salt-treated plants more than in non-primed plants. Moreover, JA priming might increase the efficiency of the photosynthesis process through the regulation of g_s_, CO_2_ transport rates, and Chl biosynthesis. This was evident in the enhanced Fv/Fm ratio and the increased growth and yields of wheat plants treated with both JA and salts. Still, JA stimulates antioxidant defense machinery (e.g., tocopherols, phenolics, SOD, and APX) that scavengers the ROS accumulation, improving wheat growth in the saline stress environment.

## Conclusions

The current study demonstrated that JA priming significantly improved all plant growth parameters and yield traits of two wheat cultivars under control and saline conditions. Moroever, co-stressors (tissue dehydration, osmotic stress, and oxidative stress) encountered by plants under salinity stress were also alleviated by JA treatment. This mitigation effect can be explained by JA's ability to enhance the structural stability and functional activity of PSII, which was reflected by the performance indices (g_s_, C_i_, and T_r_). JA also increased Na^+^ exclusion and transportation in various organs by upregulating several key genes potentially involved in Na^+^ uptake, transport and sequestration *i.e. SOS1, NHX2*, and *HVP1*. The restriction of Na^+^ accumulation in organs under salt stress was correlated with salt stress tolerance in wheat. Therefore, upon subsequent exposure to salinity stress, seed priming with JA can effectively mitigate stress-responsive criteria and enhance plant tolerance to salinity stress in an eco-friendly way. In this regard, the adoption of better agricultural practices such as on-farm hormone seeds priming by agricultural sectors could significantly have a potential for environmental stress mitigation. Development of these sustainable practices will also ensure that high crop yield and quality are available to meet total demand from agriculture.

## Data availability statement

The raw data supporting the conclusions of this article will be made available by the authors, without undue reservation.

## Author contributions

MS, AE-K, KE-T, and SA: conceptualization, methodology, writing—reviewing and editing, and supervision. MS, WQ, HL, and MD: data curation, writing—original draft preparation, software, and investigation. ZU, HA, TM, SSus, VR, AE-K, IJ, SSul, ME-E, KE-T, and SA: data curation, writing—original draft preparation, methodology, visualization, and investigation. HA and HY: software and validation. All authors contributed to the article and approved the submitted version.

## Funding

This research was supported by the Strategic Academic Leadership Program of the Southern Federal University (Priority 2030). This work was also funded by Abu Dhabi Research Award (AARE2019) for Research Excellence-Department of Education and Knowledge (ADEK-007; Grant #: 21S105) to KE-T and Khalifa Center for Biotechnology and Genetic Engineering-UAEU (Grant #: 12R028) to SA. This work was also supported by the Nature Science Foundation for Excellent Young Scholars of Jiangsu Province (BK20200057) to HL.

## Conflict of interest

The authors declare that the research was conducted in the absence of any commercial or financial relationships that could be construed as a potential conflict of interest.

## Publisher's note

All claims expressed in this article are solely those of the authors and do not necessarily represent those of their affiliated organizations, or those of the publisher, the editors and the reviewers. Any product that may be evaluated in this article, or claim that may be made by its manufacturer, is not guaranteed or endorsed by the publisher.

## Supplementary material

The Supplementary Material for this article can be found online at: https://www.frontiersin.org/articles/10.3389/fpls.2022.886862/full#supplementary-material
