# Supplementary material for: Association of jasmonic acid priming with multiple defense mechanisms in wheat plants under high salt stress
Source: Front Plant Sci. 2022 Aug 16;13:886862. doi: 10.3389/fpls.2022.886862 (PMC9429808; doi:10.3389/fpls.2022.886862)
Supplement: Supplementary file 1 [file Data_Sheet_1.pdf]

Supplementary Material

Association of jasmonic acid priming with multiple defense mechanisms in wheat plants under high salt stress

Mohamed S. Sheteiwy, Zaid Ulhassan, Weicong Qi, Haiying Lu*, Hamada AbdElgawad, Tatiana Minkina, Svetlana Sushkova, Vishnu D. Rajput, Ali El-Keblawy, Izabela Josko, Saad Sulieman, Mohamed A. El-Esawi, Khaled A. El-Tarabily*, Synan F. AbuQamar*, Haishui Yang, Mona Dawood

*** Correspondence:**

Haiying Lu luhaiying@jaas.ac.cn

Khaled El-Tarabily: ktarabily@uaeu.ac.ae

Synan AbuQamar: sabuqamar@uaeu.ac.ae

Supplementary Materials

Table S1. Meteorological data of temperature (°C), Rainfall (mm) and sun radiation (h) during 2018/2019 and 2019/2020 growing seasons.

Table S2. Some physical and chemical properties of the experimental soils used in the study during 2018/2019 and 2019/2020 growing seasons.

Table S1. Meteorological data of temperature (°C), Rainfall (mm) and sun radiation (h) during 2018/2019 and 2019/2020 growing seasons.

**Months**	**Air temperature (°C)**		**Rainfall (mm)**		**Sun radiation (h)**	
	**2018/2019**	**2019/2020**	**2018/2019**	**2019/2020**	**2018/2019**	**2019/2020**
November	11.4	13.4	82.8	41.7	86.1	161.9
December	6.2	7.3	117.4	59.1	76.5	143.9
January	4.1	5.1	51.5	75.3	92.5	44.6
February	4.4	8.2	90.4	35.5	40.2	130.2
March	11.8	12.3	37.6	89.9	168.5	153.4
April	17	15.8	52.9	70.8	158.7	210.9
May	21.8	23	41.1	33.7	194.2	167.8
June	25.4	25.8	109.2	358	164.5	95.3

Table S2. Some physical and chemical properties of the experimental soils used in the study during 2018/2019 and 2019/2020 growing seasons.

**Soil properties**	**2018/2019**	**2019/2020**
Clay %	24.90	24.30
Sand %	55.60	56.10
Silt %	19.50	19.60
Soil organic matter	22.45 g kg^{-1}	22.30 g kg^{-1}
Total nitrogen	0.87 g kg^{-1}	0.89 g kg^{-1}
Aalkali-hydrolyzable nitrogen	120.96 mg kg^{-1}	115.75 mg kg^{-1}
Available phosphorus	5.44 mg kg^{-1}	5.64 mg kg^{-1}
Available potassium	58.17 mg kg^{-1}	61.25 mg kg^{-1}
pH	7.47	7.43
Electrical conductivity (EC)	4.6 dSm^{-1}	4.35 dSm^{-1}
